# New Appliances and Things Medical

**Published:** 1904-07-23

**Authors:** 


### NEW APPLIANCES AND THINGS MEDICAL.

[We shall be glad to receive, at our Office, 28 & 29 Southampton Street, Strand, London, W.C., from the manufacturers, specimens of all new preparations and appliances which may be brought out from time to time.]

### BENGER'S FOOD.

(Benger's Food, Limited, Ottree Works, Manchester.)

We have received a sample of this well-known food which has long been a favourite, and, we think, rightly, with the medical profession. For the benefit of our general readers we may, perhaps, say that this food consists of a mixture of wheat flour and pancreatic extract. In order to get the full benefit of the food the directions must be carefully followed. When this is done most of the starch originally present in the food is converted into a soluble condition by the pancreatic extract present, and, further, the proteid of the milk used to make the food is also partially digested. The result is that we have in this preparation really a predigested food.

### BILLON'S OVO-LECITHIN.

(16 Water Lane, Great Tower Street, London, E.C.)

The importance of lecithin (distearyl-cholin glycerophosphate) as a therapeutic agent in malnutrition and nervous debility is well known. This substance is contained in meat, brain-tissue, fish-roe, and especially eggs. In what way the lecithines obtained from these different sources differ we do not at present know. The readiest source of lecithin at present is eggs, and this so-called ovo-lecithin is the active principle of the preparation before us. The ovo-lecithin is present in a granular form, and can be taken in water, in which it forms an opalescent solution, or equally well as it is. It tastes slightly sweet, and perhaps this is a disadvantage. The dose is given as from one teaspoonful to a tablespoonful, but we have not been able to find any data with regard to the actual quantity of lecithin it contains. We think that it is an agreeable form in which to give this substance, and that it can be recommended.

